# Copy number variant hotspots in Han Taiwanese population induced pluripotent stem cell lines - lessons from establishing the Taiwan human disease iPSC Consortium Bank

**DOI:** 10.1186/s12929-020-00682-7

**Published:** 2020-09-04

**Authors:** Ching-Ying Huang, Ling-Hui Li, Wan-Tseng Hsu, Yu-Che Cheng, Martin W. Nicholson, Chun-Lin Liu, Chien-Yu Ting, Hui-Wen Ko, Shih-Han Syu, Cheng-Hao Wen, Zhuge Yan, Hsiang-Po Huang, Hong-Lin Su, Po-Min Chiang, Chia-Ning Shen, Hsin-Fu Chen, B. Lin Ju Yen, Huai-En Lu, Shiaw-Min Hwang, Shih-Hwa Chiou, Hong-Nerng Ho, Jer-Yuarn Wu, Timothy J. Kamp, Joseph C. Wu, Patrick C. H. Hsieh

**Affiliations:** 1 Institute of Biomedical Sciences, Academia Sinica, Taipei, 115 Taiwan; 2 School of Pharmacy, College of Medicine, National Taiwan University, Taipei, 100 Taiwan; 3 Bioresource Collection and Research Center, Food Industry Research and Development Institute, Hsinchu, 300 Taiwan; 4 Stanford Cardiovascular Institute, Stanford University School of Medicine, Stanford, CA 94305 USA; 5 Graduate Institute of Medical Genomics and Proteomics, College of Medicine, National Taiwan University, Taipei, 100 Taiwan; 6 Department of Life Sciences, National Chung-Hsing University, Taichung, 402 Taiwan; 7 Institute of Clinical Medicine, National Cheng Kung University, Tainan, 701 Taiwan; 8 Genomics Research Center, Academia Sinica, Taipei, 115 Taiwan; 9 Institute of Cellular and System Medicine, National Health Research Institutes, Zhunan, 350 Taiwan; 10 Institute of Pharmacology, School of Medicine, National Yang-Ming University, Taipei, 112 Taiwan; 11 Department of Obstetrics and Gynecology, College of Medicine and the Hospital, National Taiwan University, Taipei, Taiwan; 12 Stem Cell and Regenerative Medicine Center, University of Wisconsin-Madison, Madison, WI 53705 USA

**Keywords:** Human induced pluripotent stem cell, Cell differentiation, Stem cell bank, Drug screening, Copy number variant, Hotspot

## Abstract

**Background:**

The Taiwan Human Disease iPSC Service Consortium was established to accelerate Taiwan’s growing stem cell research initiatives and provide a platform for researchers interested in utilizing induced pluripotent stem cell (iPSC) technology. The consortium has generated and characterized 83 iPSC lines: 11 normal and 72 disease iPSC lines covering 21 different diseases, several of which are of high incidence in Taiwan. Whether there are any reprogramming-induced recurrent copy number variant (CNV) hotspots in iPSCs is still largely unknown.

**Methods:**

We performed genome-wide copy number variant screening of 83 Han Taiwanese iPSC lines and compared them with 1093 control subjects using an Affymetrix genome-wide human SNP array.

**Results:**

In the iPSCs, we identified ten specific CNV loci and seven “polymorphic” CNV regions that are associated with the reprogramming process. Additionally, we established several differentiation protocols for our iPSC lines. We demonstrated that our iPSC-derived cardiomyocytes respond to pharmacological agents and were successfully engrafted into the mouse myocardium demonstrating their potential application in cell therapy.

**Conclusions:**

The CNV hotspots induced by cell reprogramming have successfully been identified in the current study. This finding may be used as a reference index for evaluating iPSC quality for future clinical applications. Our aim was to establish a national iPSC resource center generating iPSCs, made available to researchers, to benefit the stem cell community in Taiwan and throughout the world.

## Background

In 2006, Yamanaka and his colleagues discovered that the combination of four specific transcription factors, Oct4, Sox-2, Klf4 and c-Myc, can reprogram mouse, and later human, fibroblasts into pluripotent stem cells capable of being reprogrammed into any cell type of the three germ layers. These newly derived cells were later termed induced pluripotent stem cells (iPSCs). With the possibility of differentiating iPSCs into different somatic cell types, the discovery holds great potential as a tool for studying human disease, drug development, and cell therapy [1, 2].

Over the past decade, there has been significant progress in realizing the potential of iPSC technology. Numerous iPSC-based studies have achieved significant breakthroughs in understanding human diseases and have begun translating research from the laboratory to the clinic, for example, in the treatment of age-related macular degeneration, spinal cord injury, and type 1 diabetes [3–5]. Despite iPSC translational studies in clinical trials, genome stability and the associated downstream problems, such as tumorigenesis, remain a key issue. Single nucleotide variations (SNV), structural variation such as copy number variations (CNV) or loss of heterozygosity (LOH), which may be inherited from donor cells, generated during the reprogramming process, or prolonged culture constrain the usage of iPSCs in basic research and clinical applications. Indeed, the world’s first iPSC-based treatment for age-related macular degeneration was initially cancelled upon the discovery of SNVs and CNVs in the patient’s iPSCs raising concerns about tumorigenicity [5, 6].

Further studies have shown that CNV amplification of the 20q11.21 region is the most recurrent hotspot in iPSCs and embryonic stem cells [7–10]. The genes encompassed by this region include anti-apoptotic genes, inhibitor of DNA binding 1 (ID1), BCL2-like1, and DNMT3B which is associated with pluripotency [11]. Moreover, by using high-resolution array comparative genomic hybridization to identify the unique CNV signatures for human iPSCs, Martins-Taylor et al. (2011) found that more than 25% of human iPSCs possessed recurrent CNVs at 1q31.3 and 17q21, and a human iPSC-specific CNV deletion at 8q24.3. Differences in the occurrence of genetic variation such as single nucleotide polymorphisms (SNPs) and CNV have been reported across ethnic groups, which may result in phenotypic variation and/or disease susceptibility [12]. However, whether there is a reprograming-induced genetic variation signature in iPSCs is still largely unknown.

“Han Chinese” is the largest ethnic group in the world; representing a unique population with different genetic backgrounds compared to others in the world, and most of Han Taiwanese are of Han Chinese descent. The Han Taiwanese population is particularly unique due to the diversity of its gene pool which, in part, has arisen from colonizers such as the Dutch, Portuguese and Japanese. Over the past few years, several countries have established iPSC core facilities with the aim of improving the consistency and standardization of generating new iPSC lines [13–15]. In 2015, five institutes came together to meet an unmet need in Taiwan and formed the Taiwan Human Disease iPSC Service Consortium. This project sought to establish the first, validated, high-quality iPSC bank from healthy individuals and patients in Taiwan with the aim to develop therapies that effectively and exclusively target Chinese populations. Another objective was to employ our iPSC lines to address whether there is any genetic variation associated with reprogramming. At present, the project has generated 83 iPSC lines consisting of 11 normal iPSC lines from healthy donors and 72 iPSC lines covering 21 types of diseases. These lines span inherited diseases, chromosomal disorders, heart disease, neurodegenerative disease, neuropsychiatric disorders and rare diseases such as Fabry disease and facioscapulohumeral muscular dystrophy. We provide systematically derived and comprehensively characterized iPSC lines generated using our standardized operation protocols. To this end, each cell line was fully characterized for pluripotency and the presence of de novo CNVs. When comparing de novo CNVs in our iPSC lines with 1093 control subjects, we identified novel hotspots for recurrent CNVs during the reprogramming process. Additionally, we demonstrated the ability of our normal iPSC lines to differentiate into various somatic cell types, as well as a set of normal and disease iPSCs to specifically differentiate into cardiomyocytes (CM). Furthermore, using normal CMs, we demonstrate pharmacological responses to both isoproterenol and propranolol. These cells were also successfully engrafted in vivo.

To increase the utility of these valuable resources, both normal and disease iPSCs and the results of their characterization are available from the Bioresource Collection and Research Center (http://bcrc.firdi.org.tw/). We hope that sharing our experience in establishing this iPSC bank and generating quality-controlled iPSCs to benefit future efforts in basic and clinical research of the local and international stem cell research community.

## Methods

### Reprogramming of donor cells

Donor recruitment was approved by the Institutional Review Board of Biomedical Science Research at Academia Sinica (approval number AS-IRB02–106154 and AS-IRB02–105099). All samples were collected from donors who agreed, by signed consent, to tissue donation and iPSC derivation. The reprogramming experiment was modified from a standard procedure of iPSC reprogramming [2] using CytoTune-iPS 2.0 Sendai Reprogramming Kit (Thermo Fisher Scientific).

### Detection of Sendai virus vectors and pluripotent gene expression

To ensure the removal of Sendai virus and exogenous transgenes, and expression of endogenous pluripotent markers in iPSCs, total RNA was extracted from cells of greater than 10 passages using TRIzol reagent (Invitrogen). Then reversed transcription was performed by RevertAidTM H Minus First Strand cDNA Synthesis Kit (Fermentas). The primer sets are listed in Table S1.

### Immunofluorescence staining for pluripotency markers

The PSC4-Marker Immunocytochemistry Kit (Life Technologies, Invitrogen) was used to analyze iPSC pluripotent marker expression. Following fixation and permeabilization, cells were stained with primary antibodies against OCT4, SOX2, SSEA-4 and TRA-1–60 and secondary antibodies with Alexa 594-and Alexa 488-conjugated for red and green fluorescence, respectively (Molecular Probes).

### Embryoid body formation assay

Embryoid body formation was used to confirm differentiation potential into the three germ layers in vitro. Human iPSCs were cultured in DMEM/F12 supplemented with 20% FBS in ultra-low attachment 6-well plates (Corning) for 7 days after which embryoid bodies were re-plated onto 0.1% gelatin-coated plates. Fourteen days after re-plating, cells were then fixed with 4% formaldehyde and stained with antibodies against ectodermal marker βIII-Tubulin (TUJI), mesodermal marker α-SMA, and endodermal marker, AFP (3-Germ Layer Immunocytochemistry Kit, Thermo Fisher Scientific).

### Teratoma formation assay

Cells (1 × 10^6^) were dissociated, re-suspended in 50% Matrigel (Corning) and then transplanted into the testis of NOD/SCID mice. Mice were sacrificed on week 8 after transplantation. The teratomas were harvested, fixed and then embedded in paraffin for serial sectioning and histological analysis by haemotoxylin and eosin staining to confirm differentiation potential into different germ layers.

### Karyotyping

Center for Medical Genetics of Changhua Christian Hospital in Taiwan performed karyotyping analysis. In order to induce cell cycle arrest and nuclear swelling, cells were treated with 10 μg/mL of Colcemid and 0.075 M hypotonic KCl. The samples were then fixed with Carnoy’s fixative. Metaphase chromosomes were harvested and subjected to Giemsa staining for cytogenic analysis of G-bands.

### In vitro cardiac toxicity assay

Eighty thousand human iPSC derived CMs were treated with various dosages of doxorubicin, ranging from 1μM to 0.1 M (Selleckchem) for 24 h. Cell viability was assessed by Trypan Blue exclusion assay (Sigma) and cells were counted using TetraZ cell counting kits (BioLegend). The terminal deoxynucleotidyl transferase dUTP-mediated nick-end labeling (TUNEL) method was used to detect apoptotic cells after doxorubicin treatment. ApopTag Fluorescein in situ apoptosis detection kit was used as recommended by the manufacturer (Millipore).

### Cell engraftment assay

Human iPSC derived CMs were used for cell engraftment assay after 20 days of CM differentiation. 1 × 10^6^ CMs were suspended in fetal bovine serum (Gibco) supplemented with 100 ng/mL IGF1 (Peprotech) and 0.1% of hyaluronic acid (Creative PEGWorks). Cells were injected into immunodeficient (SCID) mice; mice were sacrificed two weeks after transplantation. The graft was immunohistochemically confirmed with anti-human mitochondria antibody (Millipore, #MAB1273) and anti-Troponin I antibody (Genetex, GTX28289).

### Genomic DNA extraction

Genomic DNA was prepared using the DNeasy Blood & Tissue Kit (Qiagen) from each iPSC line and PBMCs as recommended by the manufacturer.

### Whole genome sequencing

For Illumina library preparation, double-stranded DNA was quantified with a Qubit fluorescence assay (Life Technologies). The genomic DNA was sheared with a Covaris S2 instrument. Next Generation Sequencing library preparation was carried out using the TruSeq PCR free DNA HT kit (Illumina), essentially following manufacturer’s manual. Individual DNA libraries size and concentration were measured using an Agilent 2100 bioanalyzer (Agilent), qPCR and Qubit (Life Technologies). Libraries were normalized to 4 nM and stored at − 20 °C until use. For clustering and sequencing, normalized DNA libraries were combined into 5- or 6-sample pools per flowcell in all 8 lines and clustered on a cBot cluster instrument with paired-end cluster kit V4. All flowcells were sequenced on the Illumina HiSeq2500 sequencer using SBS kit V4 chemistry. In this study, we adopted the cloud-based DNA-seq analytics platform, SeqsLab (Atgenomix), to implement and accelerate the WGS best practice pipelines including data pre-processing, calling, annotation and interpretation of sequence variants [16]. Sequencing paired-end reads were mapped to the hg19 assembly of the human genome reference by using the BWA-MEM v.0.7.15 alignment algorithm [17]. Then we applied marking duplicates, pursuing local realignment, recalibrating base quality scores. The alignment files (BAM) generated from the data pre-processing were used with the following variant calling methods. SNVs and short indels were detected based on the GATK v.3.7 WGS Best Practices workflow by recalibrating base quality scores and performing indel realignment prior to SNV and indel calling, and recalibrating variant quality scores after variant calling. For structural variation discovery, we adopted DELLY2 v.0.7.6, which combines paired-end mapping information and split-read analysis for the discovery of balanced and unbalanced forms of structural variation, i.e., deletions, duplications, inversions, insertion and translocations, achieving high sensitivity and specificity throughout the genome. For somatic mutation discovery, we used GATK v.3.7 MuTect2, a somatic-specific genotyping tool with high sensitivity and specificity, for calling somatic SNVs and indels along with using COSMIC coding-mutations database v.77 in conjunction with dbSNP b147 database to adjust the threshold for evidence of the variants in the parental cells. For variant annotation, many popular databases were curated in SeqsLab, the databases were categorized into several groups such as population, genomic context, clinical context, and functional context [16]. Common variants were defined as those with a frequency > 1% in the population database (e.g., The 1000 Genomes, ExAC, HapMap); rare variants were defined as those with frequency < 1%. dbNSFP v.3.0 was used for annotation of functional perdition. Protein length change was defined as indels located in the exon.

### Healthy control subjects

The 1093 control subjects were chosen from the Han Chinese Cell and Genome Bank (HCCGB) in Taiwan. All subjects received a physical check-up and questionnaire screening to exclude abnormal physical conditions and mental illness [18]. These individuals’ CNV results served as controls in the CNV hotspot identification study.

### CNV hotspots and polymorphic CNV identification

Affymetrix genome-wide human SNP array 6.0 (Affymetrix) was used for genome-wide CNV screening. The experiment was conducted according to the manufacturer’s instruction by the National Center for Genome Medicine (Academia Sinica, Taiwan). All samples passed genotype quality control and genotype and copy number state of each probe was called using Affymetrix Genotyping Console software v.4.1. Sample identity was confirmed by matching the genotype data of the iPSC lines and their parental cell samples. Regions that contained at least ten consecutive probes with the same direction of copy number change were defined as having CNVs. We filtered out centromeric regions (hg19, UCSC), antibody variable regions, T cell receptor loci, pseudoautosomal regions and X-transposed-region. In this study, only CNV regions larger than 100 kb were included for further analysis. Paired analysis mode in Genotyping Console software was applied to identify iPSC-specific CNVs. The output was manually curated. CNVs identified in iPSC and overlapped more than 50% with any CNV region identified in their parental cells were excluded in order to eliminate inherited CNVs. To determine the frequency of iPSC-specific CNV regions identified in this study in the general population, iPSC-specific CNV loci were compared to control subjects. Loci that overlapped < 10% with CNVs in control subjects were included for analysis. iPSC-specific CNV hotspots were defined as the frequency of the CNVs in this study higher than 5% in the iPSC samples but not in the germline cells and rare in control subject group (< 0.2%). Likelihood Ratio Chi-square test was used to compare the difference in the CNV region between the iPSC sample and the control group, and a Bonferroni correction was used to adjust the *P* value. *P* ≤ 0.00017 is considered as significant.

To identify polymorphic CNV regions with frequent rearrangment, CNV regions were first identified in either parental cells or iPSC lines. Those regions with a frequency of occurrence > 5% in the total samples were selected. Using the Likelihood Ratio Chi-square test, the CNV regions present in parental cells and iPSCs were compared to 1093 control subjects. Subsequently, the CNV regions were manually examined to determine whether the region was only present in either the parental cells or paired iPSC line from all individuals. A CNV region was defined as a polymorphic CNV with frequent rearrangement if the mutually exclusive events were identified in more than 10 subjects.

For CNV burden assay, paired t-test was used to determine the association of total CNV count with reprogramming. Annotation of genes located in the CNV regions was according to UCSC genes (NCBI37/hg19).

### Cardiac differentiation and purification

iPSCs were detached using Accutase (Innovative Cell Technologies) and approximately 1 × 10^5^ cells were replated into Matrigel-coated 6 well plates. Once cells reached ~ 80% confluency, cells were treated with 6 μM CHIR99021 (Selleckchem) in RPMI/B27 insulin-free medium to induce mesoderm differentiation for 48 h. Then, the culture medium was replaced with RPMI/B27 insulin-free medium for 24 h. After that, cells were treated with 5 μM IWR-1 (Sigma) for 48 h. At day 5, the medium was changed to remove IWR-1. Cells were then cultured in RPMI/B27 medium at day 7. Thereafter the medium was changed every other day until cells started to beat. Cardiac purification was performed through glucose starvation; cells were cultured in glucose-free RPMI/B27 for 4 days to increase the purity of cardiomyocytes.

### Endothelial cell differentiation

To generate hiPSC-derived endothelial cells, we implemented a growth factor based protocol according to previous report [19].

### Neural induction and neuronal maturation

When iPSCs reach 80% confluence, cells were treated with 1 mg/ml Dispase II (Roche) and harvested by scraping (Day 0). The detached cell clumps were dissociated into 200–300 μm clusters and transferred to non-coated Petri dishes for 48 h. The differentiation medium was DMEM/F12 (Invitrogen) supplied with 20% knockout serum replacement (KSR, Invitrogen), 1 mM, non-essential amino acids (NEAA, Invitrogen), 2 mM glutamate (Invitrogen) and 100 μM 2-mercaptoethanol (Invitrogen). On Day 2 of differentiation, the cell culture medium was replaced to DMEM/F12 supplied with 1% N2 supplement (Invitrogen), 1 mM NEAAs and 2 mM glutamate. The BiSF induction factors, including 10 ng/mL recombinant human FGF-2 (rh-FGF2, R&D Systems), 10 μM SB431542 (Sigma-Aldrich) and 0.5 μM BIO (Sigma-Aldrich), were added on Day 2 for 48 h. From Day 0, 10 μM Y27632 (Cayman Chemical) was provided until the end of BiSF treatment. On Day 4, the neural induction medium was removed, and the cells were cultured in neurobasal medium (Invitrogen) with 1% N2 supplement and 10 ng/mL rh-FGF2. After neural induction, the cells were dissociated into small clumps by Accutase and seeded on 1% Geltrex (Invitrogen)-coated cell culture dishes for neural maturation. Following differentiation, NPC purity was determined by immunocytochemistry staining with Nestin antibody (Biolegend, #839801).

### Pancreatic differentiation

Human iPSCs were grown on a vitronectin-coated plate, and maintained in a chemically defined medium (CDM) supplemented with Activin A (10 ng/mL). CDM medium contains 250 mL DMEM/F12 (GIBCO) and 250 mL IMDM (GIBCO) mixed 1:1, 5 mL concentrated lipids (GIBCO), 20 uL Mercapto-thio-Glycerol (Sigma), 350 uL Insulin (ROCHE), 250uL Transferrin (ROCHE), 5 mL Pen/Strep (GIBCO), and 0.5 g Polyvinylalcohol (SIGMA). Differentiation was carried out by induced growing iPSCs in CDM-PVA + Activin-A (100 ng/mL), BMP4 (10 ng/mL), bFGF (20 ng/mL) and LY (10 mM) (AFBLy). The CDM-PVA AFBLy cocktail was changed daily until Day 3. Thereafter, the differentiation method was according to [20, 21]. Immunocytochemistry was further performed on iPSC derived cells with pancreatic and duodenal homeobox 1 antibody to confirm differentiation efficiency.

### Hepatic differentiation

The step-wise method to produce hepatocytes initiates from differentiating human iPSCs into definitive endoderm, which subsequently patterns into anterior endoderm and further developed into hepatic endoderm. The differentiation method was according to [21, 22]. Hepatic cells were further matured to produce cells expressing the hepatic specific protein albumin (ALB). Hepatic differentiation efficiency was examined by immunocytochemistry staining with albumin antibody (R&D System, #MAB1455).

### Granulosa cell differentiation

For granulosa cell differentiation, human iPSCs were manually split and plated onto ultra-low attachment multiwell plates (Corning) to form embryonic bodies (EBs). Cells were cultured in maintenance medium (80% DMEM/F12, 20% knockout serum replacement, 4 ng/mL bFGF, 0.1 mM -mercaptoethanol, and 1% NEAA (Gibco)) for 48 h. Cells were then treated with 6 ng/mL BMP4 (R&D systems) for 24 h. On day 3, cells were then cultured in 10 ng/mL BMP4, 6 ng/mL WNT3A, 6 ng/mL activin-A, and 5 ng/mL bFGF (R&D Systems) for 72 h. Then EB-derived mesoderm progenitors were transferred to gelatin-coated plates and incubated in the maintenance medium supplemented with 10 ng/mL BMP4, 5 ng/mL bFGF, and 25 ng/mL follistatin (R&D Systems) for 6 days.

## Results

### Establishment of the Taiwan human disease iPSC service consortium

**Fig. 1:**
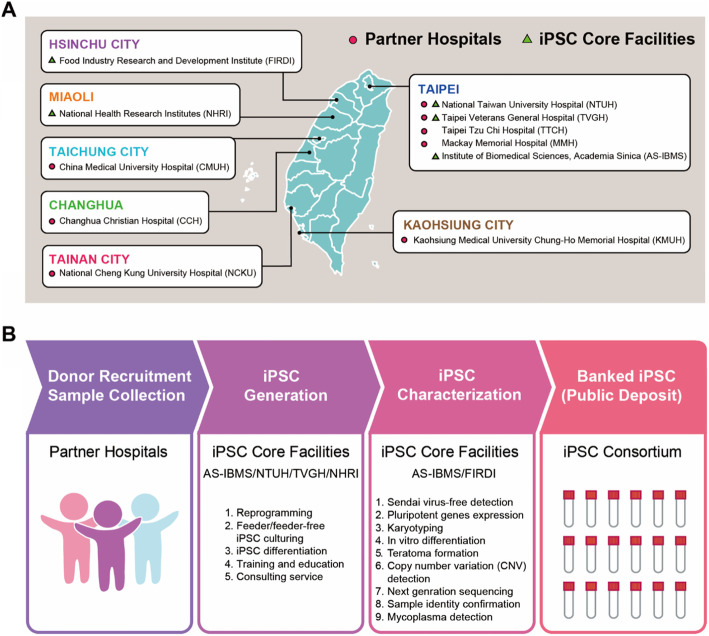
Taiwan Human Disease iPSC Consortium Bank. **a** Map of Taiwan highlighting locations of iPSC core facilities and partner hospitals. **b** Workflow of the Taiwan Human Disease iPSC Service Consortium Bank

The Taiwan Human Disease iPSC Service Consortium was established in 2015 consisting of 5 different institutes: the Institute of Biomedical Sciences, Academia Sinica (AS-IBMS), National Taiwan University Hospital (NTUH), Taipei Veterans General Hospital (TVGH), the Food Industry Research and Development Institute (FIRDI), and the National Health Research Institutes (NHRI) (Fig. 1a) and is funded by the Ministry of Science and Technology (MOST) of Taiwan. Our objective is to develop a resource for researchers both in Taiwan and abroad to provide validated and fully characterized iPSC lines which represent the Han Taiwanese population. Here, we aim to share our experience in establishing the Taiwan Human Disease iPSC Service Consortium to build the consortium facility as a resource center in Taiwan. The iPSC consortium enrolls regional hospitals across Taiwan to help identify and collect donor samples with represented diseases that fit our selection criteria to build up the Taiwan iPSC bank. Our partner hospitals include eight different organizations located throughout Taiwan; National Taiwan University Hospital (NTUH), Taipei Veterans General Hospital (TVGH), Taipei Tzu Chi Hospital (TTCH), Mackay Memorial Hospital (MMH), China Medical University Hospital (CMUH), Changhua Christian Hospital (CCH), National Cheng Kung University Hospital (NCKUH), and Kaohsiung Medical University Chung-Ho Memorial Hospital (KMUH) (Fig. 1a).

### Generation, characterization and quality control testing of Han Taiwanese iPSC lines

To ensure the consistency of the iPSC lines generated by the consortium, we established a standardized workflow across the network of sites within the consortium (Fig. 1b). All iPSC core facilities (IBMS, NTUH, TVGH and NHRI) follow a standardized protocol to handle patient samples and generate iPSCs. The Sendai virus reprogramming method has been reported as a time- and cost-effective protocol for large-scale peripheral blood mononuclear cell (PBMC)- and fibroblasts -derived iPSC biobanking [23]. Thus, all iPSC lines are generated using Sendai virus and maintained in both feeder-dependent and independent culture systems. These are subsequently sent to a centralized facility, the Bioresource Collection and Research Center (BCRC) in the FIRDI, for comprehensive characterization including, sendai virus-free detection, pluripotent gene expression, karyotyping, in vitro differentiation, and in vivo teratoma formation. Clones that successfully passed the characterization criteria were then bio-banked (Fig. 1b; Fig. S1). The only exceptions are cell lines for Turner syndrome (NTUH-iPSC-004/45,X/46,X,idic(X)(q22)), which is characterized by an abnormality in the X chromosome, and a monogenic diabetes iPSC line (TVGH-iPSC-016; 46,XY,-16,+mar [20]) who’s karyotypic abnormalities were inherited from the original donor cells (Fig. S2). Donors for the disease iPSC lines were selected based on the following criteria: patients with monogenic, polygenic, or chromosomally-inherited diseases or variants of unknown significance. In total, 76 Han Taiwanese donors were recruited, generating 83 lines. There were 11 normal lines and 72 disease lines, covering 21 diseases. Several of the disease lines carry mutations that are of high incidence in Taiwan. There were one to three patients for each disease type, with at least three clones generated for each patient. Donor gender, age, ethnicity, and known variants were also recorded. There were 43 females and 40 males in our iPSC bank (Table S2). These iPSC lines are available as a tool for research and development in Taiwan and abroad.

### Identification of genomic variation in normal Han Taiwanese iPSC lines

**Fig. 2:**
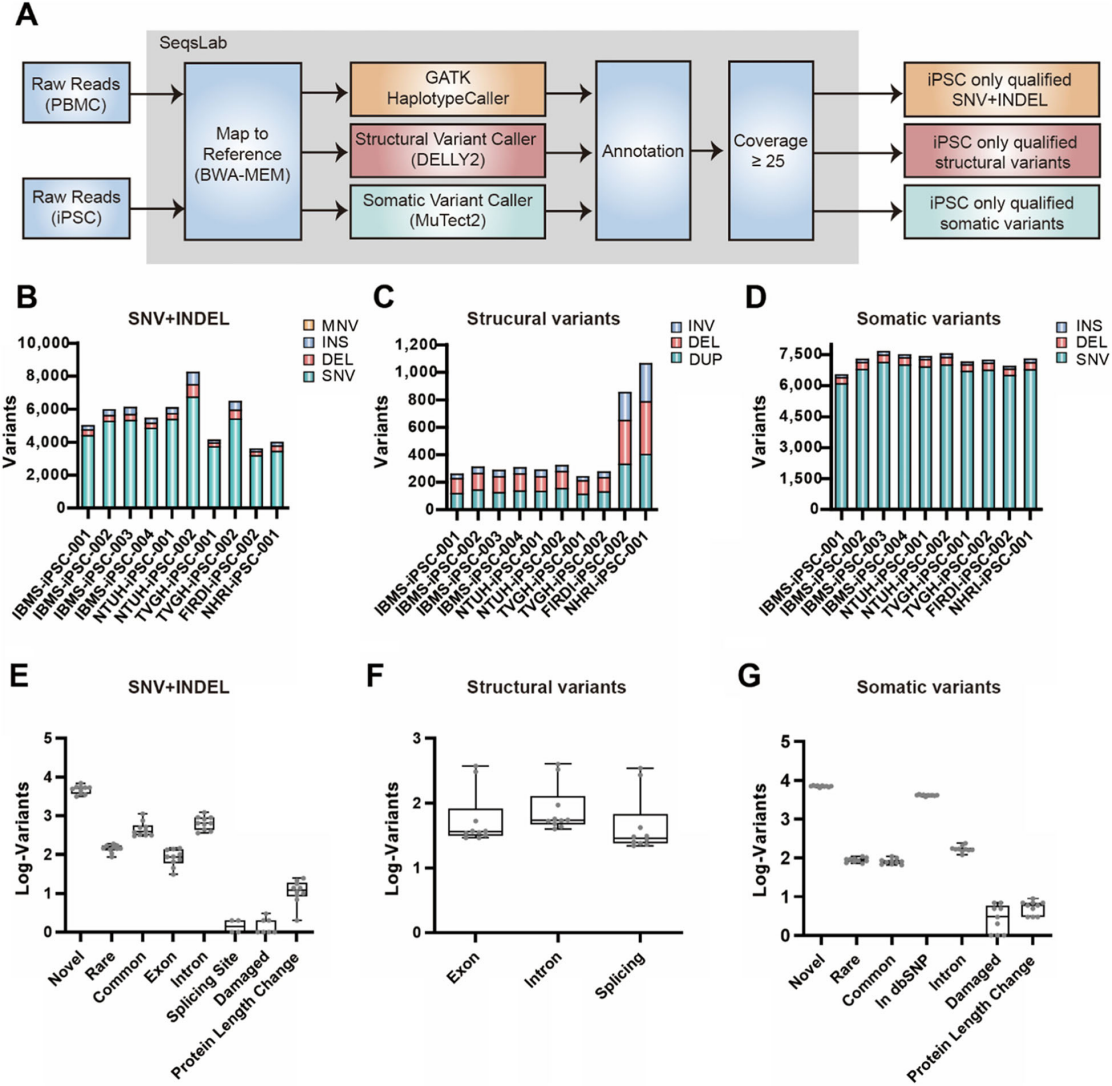
Characterization of variants generated during reprogramming in 10 normal iPSC lines. **a** A flowchart describing the filtering strategy used to obtain SNVs, structural variants and somatic variants specific to reprogrammed cell line using GATK HaplotypeCaller, DELLY2 and MuTect2. **b**-**d** The number of single nucleotide variants (SNV), deletions (DEL), insertion (INS) and multi-nucleotide variations (MNV) of iPSC-specific qualified variants among iPSCs identified by **b** GATK HaplotypeCaller, **c** DELLY2, and **d** MuTect2. **e-g** Distribution of variant categories of iPSC-specific qualified variants among iPSCs identified by **e** GATK HaplotypeCaller, **f** DELLY2, and **g** MuTect2. Data in **e**, **f**, and **g** are represented as mean ± SEM

We assessed the DNA integrity of our iPSCs by monitoring genomic variation events resulting from the reprogramming process. To this end, we conducted whole genome sequencing on ten pairs of our normal iPSC lines along with their parental cells. SeqsLab bioinformatics software (Atgenomix Inc., https://www.biorxiv.org/content/early/2017/12/27/239962) was used to streamline the genome sequencing secondary and ternary data analyses, adapted from GATK Best Practices and based on ACMG Standards and Guidelines for the interpretation of genomic variants. The data pre-processing, variant calling, variant annotation, and quality control workflows adapted are depicted in Fig. 2a. GATK3 HaplotypeCaller, DELLY2 and GATK3 MuTect2 were utilized to call different types of variants within these lines and their parental cells, such as SNVs, short insertions and deletions (indels), and structural variations. Somatic mutations were also evaluated by a Tumor-Normal variant calling method, where these iPSC lines were compared with their corresponding parental cells (PBMCs or fibroblasts) in an analogous manner to which tumor cells are compared to normal tissue to identify variants in tumor cells [24]. Variants with coverage of less than 25X and inherited variants were excluded from downstream analysis. GATK3 HaplotypeCaller was used to call SNV, short indels, and multi-nucleotide variations in ten pairs of normal iPSC-parental cells. The number of iPSC-specific variants identified across all iPSC lines ranged from 3590 to 8127 with an average of 5497. Overall, the distribution of different types of variants called by GATK3 HaplotypeCaller among all samples were similar (Fig. 2b, and Table S3). Further, classification of these variants based on population allele frequency, common variants were defined as those with a frequency ≥ 1% in the population database (The 1000 Genomes, ExAC, and HapMap); rare variants were defined as those with frequency < 1% in the population database. dbNSFP v.3.0 was used for annotation of functional perdition. Protein length change was defined as indels located in the exon. Classification of these variants based on population allele frequency, the locus in chromosomal regions, functional prediction, and sequence effect indicated no functional differences among samples (Fig. 2e). To investigate iPSC-specific pathogenic variants, GATK3 MuTect2 was utilized in accordance with the 2015 ACMG guidelines (Standards and Guidelines for the Interpretation of Sequence Variants). The number of variants identified using GATK3 MuTect2 Tumor-Normal pipeline across all iPSC lines ranged from 6523 to 7646. Based on the same classification criteria above, a similar trend in variant distribution was found in that there was no functional difference among samples (Fig. 2d). Variants affecting coding DNA sequence and the encoded protein (indels in exonic regions) were found in a small number ranging from 3 to 9 in each iPSC line (Fig. 2g). Characterization and annotation of these indicated that none were tumorigenic-related. DELLY2, a structural variant calling tool, was used to identify duplication, insertions, and deletions greater than 300 bp. The average number of iPSC-specific structural variants was 420 and was similar across all iPSC lines except for two iPSC lines which contained more structural variants (FIRDI-iPSC-002 and NHRI-iPSC-001). In general, the number of variants ranged from 241 to 1061 (Fig. 2c) and showed a uniform pattern in type and location (Fig. 2c and f). We also investigated whether there was any common genomic variation among these iPSC lines; we did not find any significant variation that was shared among the 10 iPSC lines using the three different calling methods.

### Identification of CNV hotspots in Han Taiwanese iPSC lines using Affymetrix genome-wide human SNP Array

**Fig. 3:**
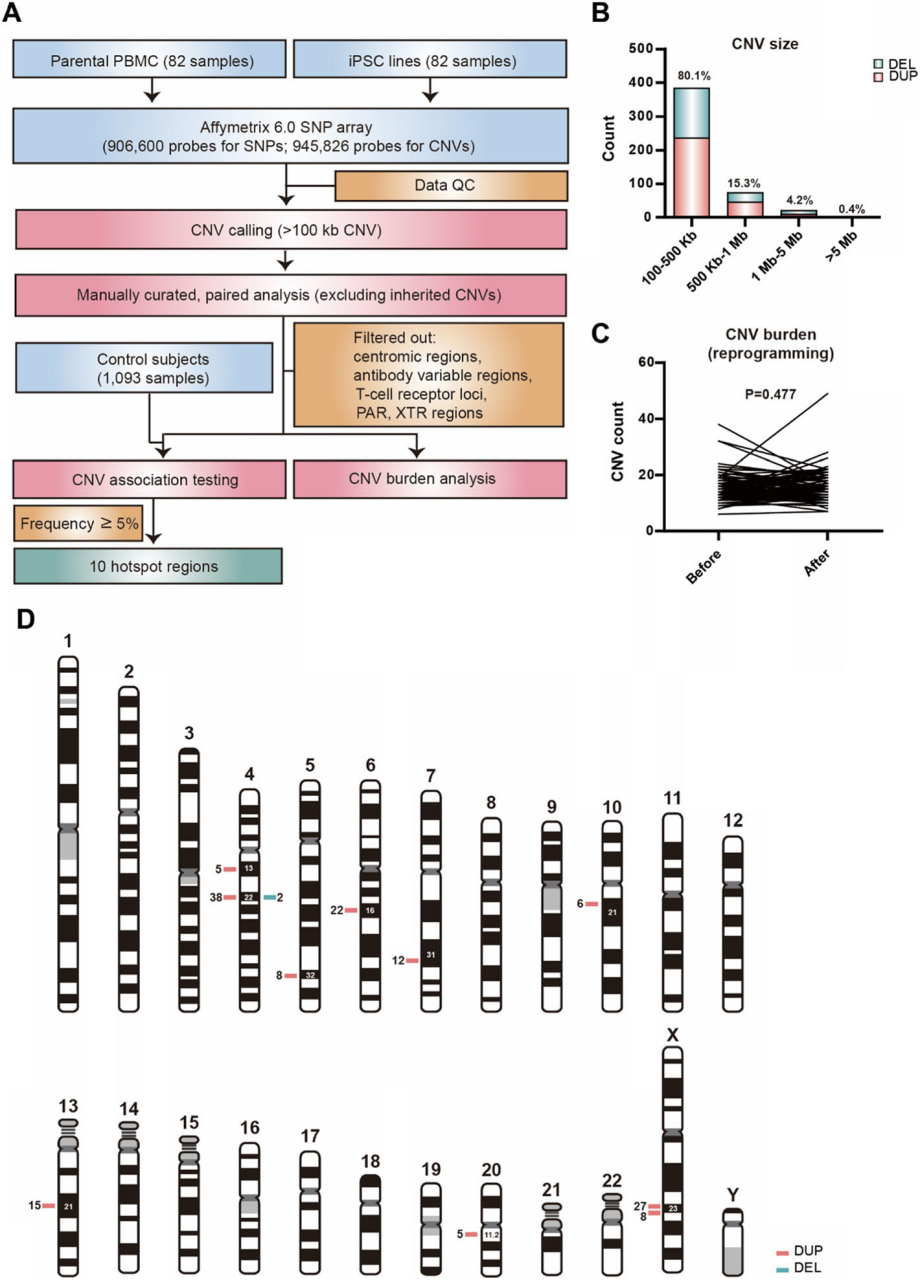
Characteristics of CNVs induced by reprogramming. **a** Workflow of CNV identification. **b** Distribution of the size of CNVs among iPSCs induced by reprogramming. **c** CNV burden analysis of cells before and after iPSC reprogramming **d** Chromosomal distribution of rearrangement of hotspot regions of iPSC-specific CNVs from 82 iPSC lines

To examine whether the reprogramming process could induce de novo CNV generation and to identify whether there are any recurrent CNV hotspots associated with the reprogramming process, we analyzed 83 iPSC lines and their parental cells using the Affymetrix Genome-Wide Human SNP Array 6.0 which contains more than 906,600 probes for SNPs, and 945,826 probes for CNVs. All samples passed the genotyping quality control test (average sample call rate = 99.24%). The pipeline for CNV filtering and annotation is summarized in Fig. 3a. CNVs that were either generated during or after the reprogramming process at autosomes and sex chromosomes were analyzed by comparing CNVs identified in iPSC lines and respective parental cells. We excluded all inherited CNVs and filtered out centromeric regions, antibody variable regions, T-cell receptor loci, pseudoautosomal regions and X-transposed-region and Turner Syndrome samples. CNVs with length larger than 100 kb were included for further analysis. CNVs located in the same loci among samples were identified using 10% reciprocal overlap cutoff. In total, we identified 168 iPSC-specific CNV loci (Table S4) from 82 iPSC samples. The details of iPSC-specific CNV regions among various iPSC lines are listed in Table S5. Notably, the length of the majority (80.1%) of CNVs was less than 500 Kb, 15.3% of CNVs were 500 Kb-1 Mb and 4.2% of CNVs were 1 Mb–5 Mb. Only 2 CNVs were larger than 5 Mb (contained in NTUH-iPSC-010 and TVGH-iPSC-024 iPSC lines; Fig. 3b). All lines were assessed for CNV burden before and after reprogramming. There was no increased burden in the iPSC lines compared to their respective parental cells (*P* = 0.477; Fig. 3c). To identify any iPSC-specific CNV hotspot regions associated with reprogramming, the rates of CNV at autosomes and sex chromosomes were compared between all iPSC lines and 1093 control subjects (525 males, 568 females) from Taiwan. CNV regions on autosomes were analyzed in all samples whereas CNV regions on sex chromosomes were analyzed by gender. iPSC-specific hotspots were defined as CNV regions with a frequency of occurrence > 5% in iPSC lines but not found in their paired parental cells and the rates of such CNV in control subjects were less than 0.2%. These hotspots were significantly associated with reprogramming. We successfully identified 10 iPSC-specific CNV hotspots located at chromosomes 4, 5, 6, 7, 10, 13, 20 and X, with 91% of the CNV hotspots being duplications (Fig. 3d and Table 1). Most of the cell sources for reprogramming in our bank are PBMCs, as PBMCs are a heterogeneous mixture of cell types. To confirm whether these hotspots can be found in iPSC lines derived from fibroblasts, we analyzed all subclones of our fibroblast-derived iPSC lines (3 iPSC lines), and found that some fibroblast-derived subclones contain the same CNV hotspot regions. For example, duplication of Chr4q22.1-q22–2 was found in all 4 subclones of IBMS-iPSC-031 and 3 subclones of TVGH-iPSC-025. Moreover, copy number gain within ChrXq23 was identified among all 4 IBMS-iPSC-031 subclones (Table S5). To determine if there are polymorphic CNV regions with frequent rearrangement during reprogramming, CNV regions with a frequency of occurrence > 5% in iPSCs and parental cells but not always existing in paired samples were selected. Using the Likelihood Ratio Chi-square test, the CNV regions present in parental cells and iPSCs were compared to the 1093 control subjects. Seven CNV regions (chromosome 1, 4, 7, 8, 14, 15 and 16) were identified as common within the iPSCs or parental cells, and control group (P > 0.05). The data showed that these 7 CNV regions are “polymorphic” CNV regions in this population, and suggested that rearrangement of these polymorphic CNV regions occurred frequently during the reprogramming process. Detailed information about these polymorphic CNV regions is listed in Table S6. These results indicated that the reprogramming process induced rearrangement of 10 CNV hotspot regions and 7 polymorphic CNV regions in the general population of Taiwan. When using the functional annotation tool DAVID for clustering of genes located within CNV hotspots and polymorphic CNVs, the result revealed that most genes were clustered in signaling peptide, secreted, and extracellular regions. We did not identify any genes within these regions which have been associated with tumorigenesis.

**Table 1:** List of genes at the hotspot regions of iPSC-specific CNVs

				iPSCs (***n*** = 82)		Control (***n*** = 1093)			
Chr	Start	End	Locus	Duplication	Deletion	Duplication	Deletion	***P*** value	Genes Contained
4	62690392	62945462	q13.1	5	0	1	0	< 0.0001	ADGRL3, ADGRL3-AS1
4	92930866	94220988	q22.1–q22–2	38	2	0	1	< 0.0001	GRID2, LNCPRESS2
5	147202104	147449067	q32	8	0	0	0	< 0.0001	SPINK5, SPINK1, SCGB3A2, C5orf46
6	94291842	94849832	q16.1	22	0	0	0	< 0.0001	TSG1
7	121549706	122245008	q31.32	12	0	1	0	< 0.0001	PTPRZ1, CADPS2, AASS, FEZF1-AS1, FEZF1
10	54932474	55398702	q21.1	6	0	0	0	< 0.0001	NA
13	55695571	56471149	q21.1	15	0	0	2	< 0.0001	MIR5007
20	29620219	31558271	q11.21	5	0	0	0	< 0.0001	MYLK2, EFCAB8, BCL2L1, HM13, FRG1BP, FRG1DP, DEFB122, COMMD7, POFUT1, NOL4L, HCK, PDRG1, NOL4L-DT, MLLT10P1, DEFB115, DEFB116, DEFB119, DEFB118, DEFB121, DEFB124, DEFB123, LINC00028, REM1, ID1, HM13-AS1, MIR3193, COX4I2, TPX2, ABALON, DUSP15, FOXS1, TTLL9, MIR7641–2, CCM2L, XKR7, PLAGL2, TM9SF4, TSPY26P, KIF3B, MIR1825, ASXL1, LOC101929698, C20orf203, DNMT3B, MAPRE1
X	112880475	113795367	q23	27	0	0	0	< 0.0001	XACT
X	114766295	114897324	q23	8	0	0	0	< 0.0001	PLS3,PLS3-AS1

* For CNVs at X-chromosome, we only compare female iPSC (*n* = 42) and female controls (*n* = 568)

### Derivation of iPSCs into different somatic lineages

**Fig. 4:**
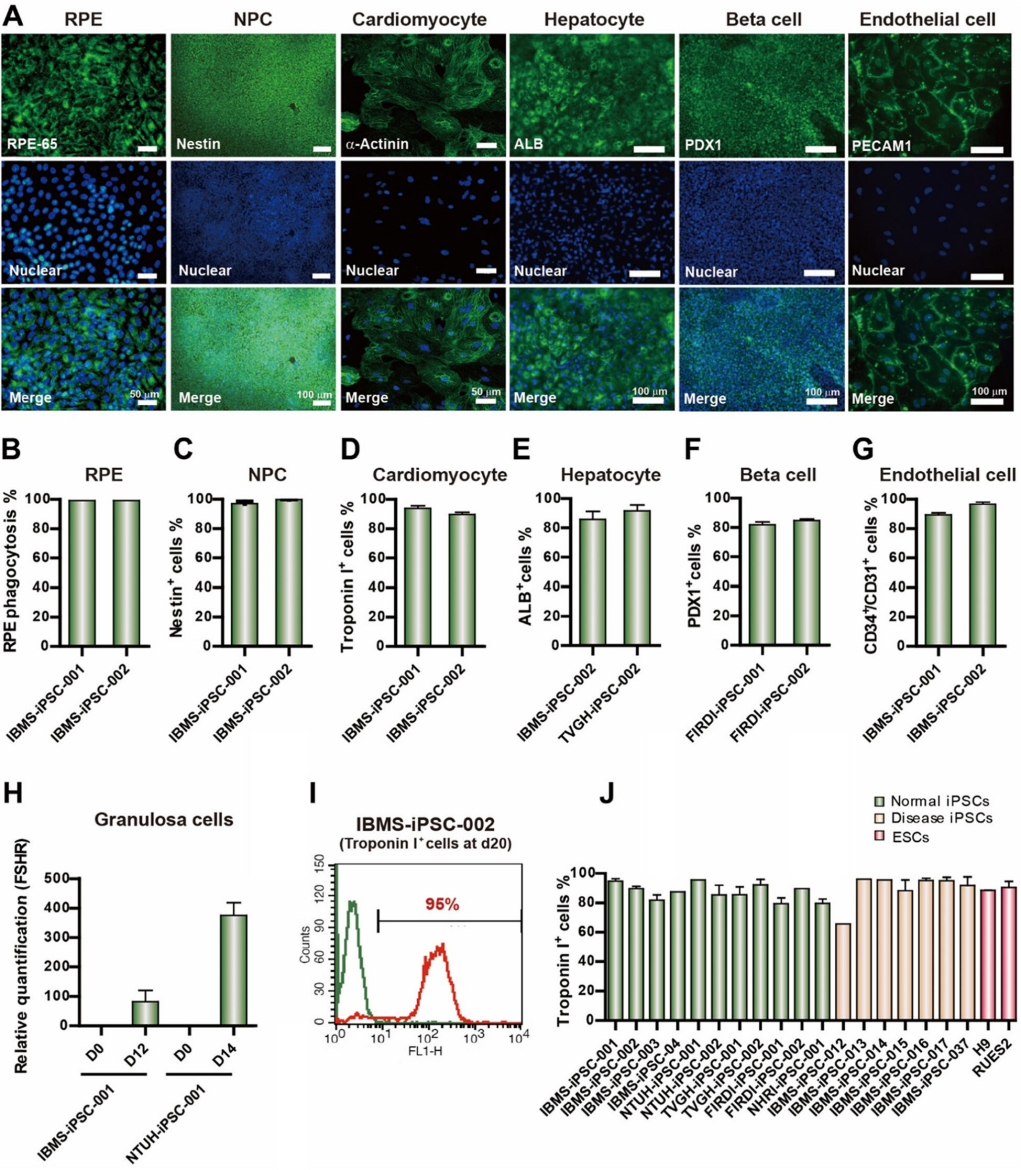
Normal iPSC lines have the ability to differentiate into different germ layer cells. **a** Immunofluorescence of various markers of normal iPSC derived cells: retinal pigment epithelium (RPE) marker RPE65, neural progenitor cells (NPC) specific antibody nestin, cardiac specific marker α-actinin, hepatic cell-specific markers albumin (ALB), β-cells precursor marker pancreatic and duodenal homeobox 1 (PDX1), endothelial cell marker PECM1. **b-g** Quantification of flow cytometry for the percentage of cells expressing **b** RPE65, **c** nestin, **d** α-actinin, **e** ALB, **f** PDX1, **g** PECM1. **h** Normal iPSC-derived cells express granulosa cell marker FSHR. **i** Flow cytometry analysis of troponin-I positive cells at 20 days after cardiomyocyte differentiation. **j** Quantification of flow cytometry data showing percentage of troponin-I positive cells in various normal and disease iPSC, and embryonic stem cell (ESC) lines undergoing cardiac differentiation. Data are represented as mean ± SEM

To assess the utility of our iPSC lines, we differentiated at least two of our normal iPSC lines into various somatic cell types, such as retinal pigment epithelium, neural progenitor cells, cardiomyocytes (CM), hepatocytes, pancreatic cells, endothelial cells and granulosa cells. Immunofluorescence was employed to verify the expression of specific markers such as the retinal pigment epithelium marker RPE65, neural progenitor cell specific marker nestin, cardiac specific marker α-actinin, hepatic cell specific marker albumin, β-cell precursor marker pancreatic and duodenal homeobox 1, and endothelial cell marker PECAM1, in their respective differentiated cell type (Fig. 4a). Quantification of cells expressing each marker is shown in Fig. 4b-g. iPSCs were also differentiated into germ-like cells, which showed mRNA expression of follicle-stimulating hormone receptor in the granulosa cell stage at day 12 and 14 after differentiation (Fig. 4h). Together, these data show that our cell lines have the potential to differentiate into multiple cell types.

### iPSC-derived cardiomyocytes respond to doxorubicin and engraft into mouse myocardium

We next sought to determine whether our normal and disease iPSC lines could be used for disease modeling, drug screening and clinical use. To this end, we differentiated 11 normal and 7 disease iPSC lines into CMs. The percentage of troponin-I positive cells was confirmed by flow cytometry on day 20 after cardiac differentiation (Fig. 4i). Quantification of troponin-I positive cells across each iPSC line is shown in Fig. 4j. To verify appropriate drug responses, we first assessed the effects of either the β-adrenergic agonist isoproterenol or the beta-blocker propranolol. The iPSC-derived CMs showed a dose-dependent response to both drugs, namely, increased beating frequency upon treatment with isoproterenol and decreased beating frequency with propranolol (Fig. S3A). The EC_50_ for isoproterenol and propranolol were 0.37 μM and 3.6 μM. We then tested the effect of 24 h exposure of increasing doses of doxorubicin, known to have cardiotoxic effects on CMs derived from two of our normal iPSC lines (IBMS-iPSC-002 and FIRDI-iPSC-002). All CMs showed a dose-dependent cardiotoxicity to doxorubicin with an IC50 of 1.9 μM and 13.6 μM (Fig. S3B). Furthermore, doxorubicin-induced cardiotoxicity was confirmed by TdT-mediated dUTP nick end-labeling (TUNEL) assay. TUNEL-positive cells were observed at 24 h after iPSC-derived CMs were treated with 1μM doxorubicin (Fig. S3C). Finally, we also examined the ability of our iPSC-derived CMs to engraft into the mouse myocardium through direct injection of 1 X 10^6^ IBMS-iPSC-001-derived CMs into the myocardium of nude mice. A schematic diagram of the cell engraftment assay is shown in Fig. S3D. Cells that successfully engrafted were identified at 2 weeks post-injection using a human-specific mitochondrial antibody (Fig. S3E) whereas the surrounding myocardial area was identified through either α-myosin heavy chain (MHC) or cardiac Troponin I antibodies. The results (Fig. S3E) showed clear engraftment of iPSC-derived CMs into the mouse myocardium which indicates the clinical potential of these cells.

## Discussion

The current report details the experience of establishing a large-cohort iPSC bank representing the genetic background of the general population in Taiwan and the identification of CNV hotspots during the reprogramming process. Since its discovery, iPSC technology has become a powerful tool for understanding the correlation between patient genotype and phenotype and recently has shown promising clinical applications [25]. In order to provide high-quality fully characterized iPSCs, the Taiwan Human Disease iPSC Consortium Bank has developed strict standard operating protocols to generate and culture each iPSC line to ensure consistency across our four iPSC core facilities. All iPSC lines were generated using Sendai virus non-integrating method. iPSCs with ES-like morphology were selected and tested for existence of the Sendai virus. Subsequent to that, standard characterization of each line included examination of cell morphology, pluripotency assessment, karyotyping, and in vitro and in vivo differentiation. We also incorporated microbiology contamination testing, genome-wide SNP and CNV detection, and cell identity confirmation (STR-PCR and SNP genotyping) in our characterization protocol. Currently, the Taiwan iPSC bank has collected and generated more than 83 fully characterized normal and disease iPSC lines spanning 21 diseases. Our iPSC bank recruited patients carrying monogenic and polygenic disorders; variants of unknown significance, chromosomally-inherited diseases; and family-specific variation with several carrying mutations that are of high incidence in Taiwan. For example, the Taiwan Human Disease iPSC Consortium Bank holds iPSC lines which carry the α-galactosidase mutation c.936 + 919G > A in Fabry disease patients. Additionally, G2385R polymorphism of the LRRK2 gene which is a known risk factor variant for Parkinson’s Disease (Cai et al., 2013) in the Han Chinese population, as well as the aldehyde dehydrogenases-2 SNP rs671, which occurs in approximately 45% of the population in Taiwan [22, 26–28]. Together, this bank holds high quality, fully characterized iPSC lines which represent the local Han Taiwanese population in Taiwan.

Previous reports have found that most CNVs are generated during early passage after cell reprogramming [15]. Due to budget scale, we used the highly cost-effective Affymetrix Genome-Wide Human SNP Array 6.0 to investigate genomic integrity. In agreement with previous studies, the overwhelming majority (97.9%) of the CNVs detected in our iPSC lines (passage 12–20) were less than 2 Mb [15]. Notably, in 8 iPSC lines, with an otherwise normal karyotype, our array data identified 10 CNVs larger than 2 Mb (2.1%), with two larger than 5 Mb. These chromosomal abnormalities were excluded when performing G-banded karyotyping as G-banded karyotyping has a limited resolution to 5 Mb–10 Mb and the risk of overlooking chromosomal abnormalities is increased when karyotyping results give poor G-banding pattern (Schrock et al., 1996). Trisomies 8 and 12 are a common karyotypic abnormality found in iPSCs and embryonic stem cells as shown in a previous study [29]. In contrast, all of our iPSC lines displayed normal karyotype except for Turner syndrome iPSC lines and a monogenic diabetes iPSC line which was also found in the parental cells and thus not due to iPSC reprogramming.

Variations in iPSCs are predominantly inherited from their parental cells [30, 31]. To highlight iPSC-specific CNV hotspots, we examined CNVs with frequency higher than 5% and only found in the iPSCs but not in the paired parental cells. Ten of these iPSC-unique CNV regions were found, and furthermore, all of them were either rare or not found in the 1093 Taiwan general population control subjects (frequency < 0.2%) (Table 1), suggesting that the reprogramming process induced these recurrent CNVs. Notably, most of the CNV hotspots we identified are copy number gain, which is unsurprising as most copy number deletions result in cell death. Intriguingly, we also identified polymorphic CNVs with frequent rearrangement during reprogramming, evident by the occurrence or disappearance of these CNVs between paired iPSC and parental cells. These results not only suggest that these CNVs might be associated with recombination hotspots during cell proliferation, but also these CNVs might be unstable from generation to generation in the population due to the frequent rearrangement nature.

Consistent with data from [32], we found that the distribution of reprogramming-induced CNVs is nonrandom as nearly 48.8% of our iPSC lines have copy number gains and losses within the Chr4q22.1-q22–2 region. This de novo CNV was also detected and verified using quantitative PCR analysis in retroviral-reprogrammed iPSCs and Sendai virus-reprogrammed iPSCs in a previous study [33]. CNV hotspot regions on chromosome 5 (Chr5q32) and 7 (Chr7q31.32) have also been reported to be generated during reprogramming [34]. In 2018, Popp et al. found that a mosaic gain in Chr20q11.21 represented a possible hotspot CNV region in two of their iPSC lines [11]. This result is consistent with our finding on duplication within Chr20q11.21 region. Genes located in the hotspot region Chr20q11.21 (ID1, BCL2L1 and HM13) were found to have minimal amplicon in ESC lines in a previous study, and BCL2L1 has been shown as a candidate for lead adaptive benefits for ESC culture [35]. Furthermore, a long noncoding RNA, XACT located in ChrXq23 duplication hotspots, has been re-expressed in human iPSCs to control human X-chromosome inactivation initiation, whereas, XACT was silenced in iPSC-derived mesenchymal stem cells [33, 36]. This result suggests that CNV hotspots may have functional significance in iPSCs. When using DAVID for functional annotation clustering of genes located within CNV hotspots, we did not find genes within these regions associated with tumorigenesis. Although we successfully identified ten CNV hotspots strongly associated with reprogramming using genome-wide SNP array, we note that there are still some technical limitations. Hybridization intensity-based CNV inference is unable to detect balanced translocations and inversions. The sensitivity of SNP array does not support the detection of low-level chromosomal mosaicism. Finally, uneven distribution of SNP probes affects the CNV evaluation. Hence, for complete characterization when surveying genomic integrity of iPSCs, both genome-wide analysis and G-banded karyotyping should be included.

## Conclusions

In summary, iPSC generation and characterization are time-consuming, expensive, and labor-intensive process. We hereby present the establishment of the first iPSC core in Taiwan, representing genetically, the general Han Taiwanese population. The implementation of standardized protocols for iPSC generation and characterization enabled consistent production of high-quality iPSCs. Several iPSC lines were obtained from normal, healthy subjects, as well as numerous iPSC lines from patients with a spectrum of representative diseases. Furthermore, the CNV hotspots induced by cell reprogramming have successfully been identified in the current study. Whether evaluation of genetic variation should be included in iPSC characterization remains unclear, however, this finding may be used as a reference index for evaluating iPSC quality for future clinical applications. We also expect that the CNV hotspots identified in this study can help to establish characterization standards for iPSC genetic integrity. Overall, these items mirror issues discussed in previous international stem cell banking initiatives [37]. With the establishment of an easily-accessible service, systematic procedures, and well-organized repository, we envisage that the core facility and iPSC bank will become an invaluable resource for both public and private research.

## Supplementary information

**Additional file 1. Figure S1.** Characterization of iPSC cell line IBMS-iPSC-002-07. (A) Reverse transcription-polymerase chain reaction (RT-PCR) analyses of Sendai-virus (SEV) and human KLF4, KLF4- OCT3/4-SOX2 (KOS), c-MYC, and GAPDH . (B) RT-PCR analyses of ESC-marker expression in the iPSC line. (C) Immunofluorescence analyses of stemness marker expression. (D) In vitro differentiation of iPSCs into the three different germ-layers by embryonic formation assay. (E) Histological staining of teratoma derived from a normal iPSC line; N: neuronal structure, G: glandular structure; C: cartilage-like structure. (F) Representative G-banded chromosomes. Karyotypes of normal iPSCs. **Figure** **S2** Karyotype analysis of the iPSC lines. (A) Karyotype of premature ovarian failure iPSC, NTUH-iPSC-004-06 (Turner disease). (B) Karyotype of parental cells of TVGH-iPSC-016, arrow indicates rearranged chromosome 16. (C) Karyotype of TVGH-iPSC-016 (monogenic diabetes iPSC), arrow indicates missing chromosome 16. **Figure S3** In vitro and in vivo functional evaluation of iPSC derived cardiomyocytes. (A) Beating frequency (beats/min) of iPSC-derived cardiomyocytes under isoproterenol or propranolol treatment. (B) The dose-response relationship for doxorubicin (DOX) treatment of iPSC derived cardiomyocytes, as evaluated by TetraZ cell counting assay. (C) TUNEL assay showing cell death 24 h after doxorubicin treatment. (D) Workflow of iPSC-CM engraftment in the mouse heart. (E) Immunostaining of the mouse myocardium engrafted with derived from human iPSC, showing engrafted of human iPSC-CMs. h-Mito: anti-human mitochondria antibody. Data are represented as mean ± SEM.

**Additional file 2. Table S1.** Primer list for RT-PCR. **Table S2.** A List of Available Normal and Disease iPSCs in the Taiwan Disease iPSC Service Consortium Cell Bank. **Table S3.** The number of SNV, DEL, INS and MNV of iPSC-specific qualified variants among iPSCs identified by GATK HaplotypeCaller. **Table S4.** List of iPSC-specific CNV loci. **Table S5.** Summary of iPSC-specific CNV loci among various iPSC lines. **Table S6.** List of genes at the “polymorphic” CNV regions strongly associated with the reprogramming process

## Data Availability

The information for normal/disease iPSC lines are available in Taiwan iPSC Consortium Cell Bank (http://ipsc.ibms.sinica.edu.tw/index.html; https://catalog.bcrc.firdi.org.tw/Welcome). All iPSC lines in this study are readily available from the BCRC cell bank (https://catalog.bcrc.firdi.org.tw/Welcome). The datasets generated during and/or analysed during the current study are available from the corresponding author on reasonable request.

## Abbreviations

AS-IBMS: Institute of Biomedical Sciences, Academia Sinica
BCRC: Bioresource Collection and Research Center
CCH: Changhua Christian Hospital
CM: Cardiomyocyte
CMUH: China Medical University Hospital
CNV: Copy number variation
FIRDI: Food Industry Research and Development Institute
HCCGB: Han Chinese Cell and Genome Bank
iPSC: Induced pluripotent stem cell
KMUH: Kaohsiung Medical University Chung-Ho Memorial Hospital
LOH: Loss of heterozygosity
Indels: Short insertions and deletions
MHC: Myosin heavy chain
MMH: Mackay Memorial Hospital
NCKUH: National Cheng Kung University Hospital
NHRI: National Health Research Institutes
NTUH: National Taiwan University Hospital
PBMC: Peripheral blood mononuclear cell
RPE: Retinal pigment epithelial cell
SNP: Single nucleotide polymorphism
SNV: Single nucleotide variations
TTCH: Taipei Tzu Chi Hospital
TUNEL: TdT-mediated dUTP nick end-labeling assay
TVGH: Taipei Veterans General Hospital
