# Supplementary material for: Copy number variant hotspots in Han Taiwanese population induced pluripotent stem cell lines - lessons from establishing the Taiwan human disease iPSC Consortium Bank
Source: J Biomed Sci. 2020 Sep 4;27:92. doi: 10.1186/s12929-020-00682-7 (PMC7487458; doi:10.1186/s12929-020-00682-7)
Supplement: Supplementary file 1 — Additional file 1. Figure S1. Characterization of iPSC cell line IBMS-iPSC-002-07. (A) Reverse transcription-polymerase chain reaction (RT-PCR) analyses of Sendai-virus (SEV) and human KLF4, KLF4- OCT3/4-SOX2 (KOS), c-MYC, and GAPDH . (B) RT-PCR analyses of ESC-marker expression in the iPSC line. (C) Immunofluorescence analyses of stemness marker expression. (D) In vitro differentiation of iPSCs into the three different germ-layers by embryonic formation assay. (E) Histological staining of teratoma derived from a normal iPSC line; N: neuronal structure, G: glandular structure; C: cartilage-like structure. (F) Representative G-banded chromosomes. Karyotypes of normal iPSCs. Figure S2 Karyotype analysis of the iPSC lines. (A) Karyotype of premature ovarian failure iPSC, NTUH-iPSC-004-06 (Turner disease). (B) Karyotype of parental cells of TVGH-iPSC-016, arrow indicates rearranged chromosome 16. (C) Karyotype of TVGH-iPSC-016 (monogenic diabetes iPSC), arrow indicates missing chromosome 16. Figure S3 In vitro and in vivo functional evaluation of iPSC derived cardiomyocytes. (A) Beating frequency (beats/min) of iPSC-derived cardiomyocytes under isoproterenol or propranolol treatment. (B) The dose-response relationship for doxorubicin (DOX) treatment of iPSC derived cardiomyocytes, as evaluated by TetraZ cell counting assay. (C) TUNEL assay showing cell death 24 h after doxorubicin treatment. (D) Workflow of iPSC-CM engraftment in the mouse heart. (E) Immunostaining of the mouse myocardium engrafted with derived from human iPSC, showing engrafted of human iPSC-CMs. h-Mito: anti-human mitochondria antibody. Data are represented as mean ± SEM. [file 12929_2020_682_MOESM1_ESM.docx]

**Copy Number Variant Hotspots in Han Taiwanese Population Induced Pluripotent Stem Cell Lines - Lessons from Establishing the Taiwan Human Disease iPSC Consortium Bank**

Ching-Ying Huang^1^, Ling-Hui Li^1^, Wan-Tseng Hsu^2^, Yu-Che Cheng^1^, Martin W. Nicholson^1^, Chun-Lin Liu^1^, Chien-Yu Ting^1^, Hui-Wen Ko^3^, Shih-Han Hsu^3^, Cheng-Hao Wen^3^, Zhuge Yan^4^, Hsiang-Po Huang^5^, Hong-Lin Su^6^, Po-Min Chiang^7^, Chia-Ning Shen^8^, Hsin-Fu Chen^5^, B. LinJu Yen^9^, Huai-En Lu^3^, Shiaw-Min Hwang^3^, Shih-Hwa Chiou^10^, Hong-Nerng Ho^11^, Jer-Yuarn Wu^1^, Timothy J. Kamp^12^, Joseph C. Wu^4^, and Patrick C. H. Hsieh^1*^

**Supplemental information**

**Additional file 1 includes:**

1. Fig. S1-S3

**Additional file 2 includes:**

1. Table S1-S6

**Supplementary figures and figure legends**


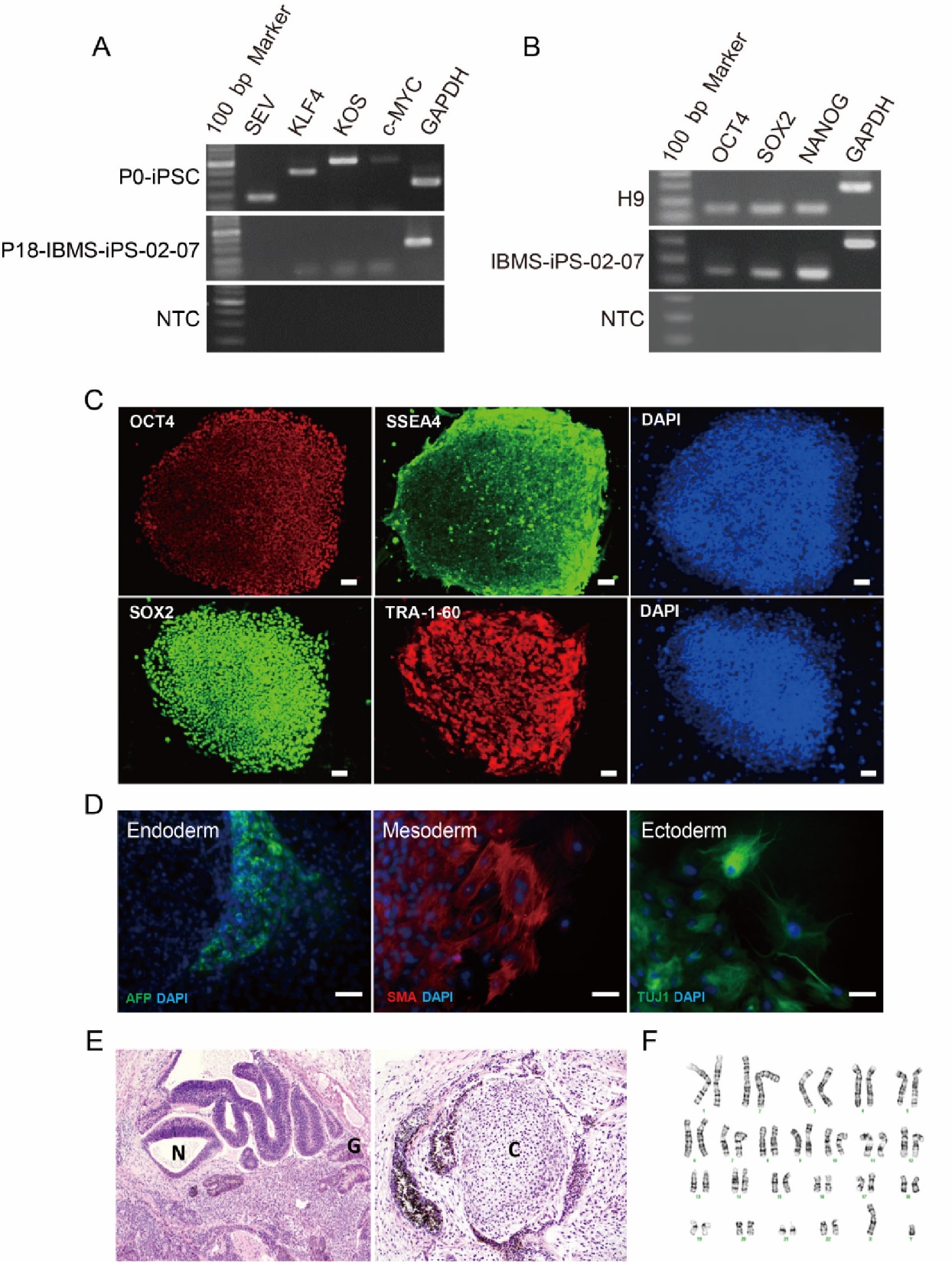


**Fig. S1 Characterization of iPSC cell line IBMS-iPSC-002-07.** (A) Reverse transcription-polymerase chain reaction (RT-PCR) analyses of Sendai-virus (SEV) and human KLF4, KLF4- OCT3/4-SOX2 (KOS), c-MYC, and GAPDH . (B) RT-PCR analyses of ESC-marker expression in the iPSC line. (C) Immunofluorescence analyses of stemness marker expression. (D) *In vitro* differentiation of iPSCs into the three different germ-layers by embryonic formation assay. (E) Histological staining of teratoma derived from a normal iPSC line; N: neuronal structure, G: glandular structure; C: cartilage-like structure. (F) Representative G-banded chromosomes. Karyotypes of normal iPSCs.


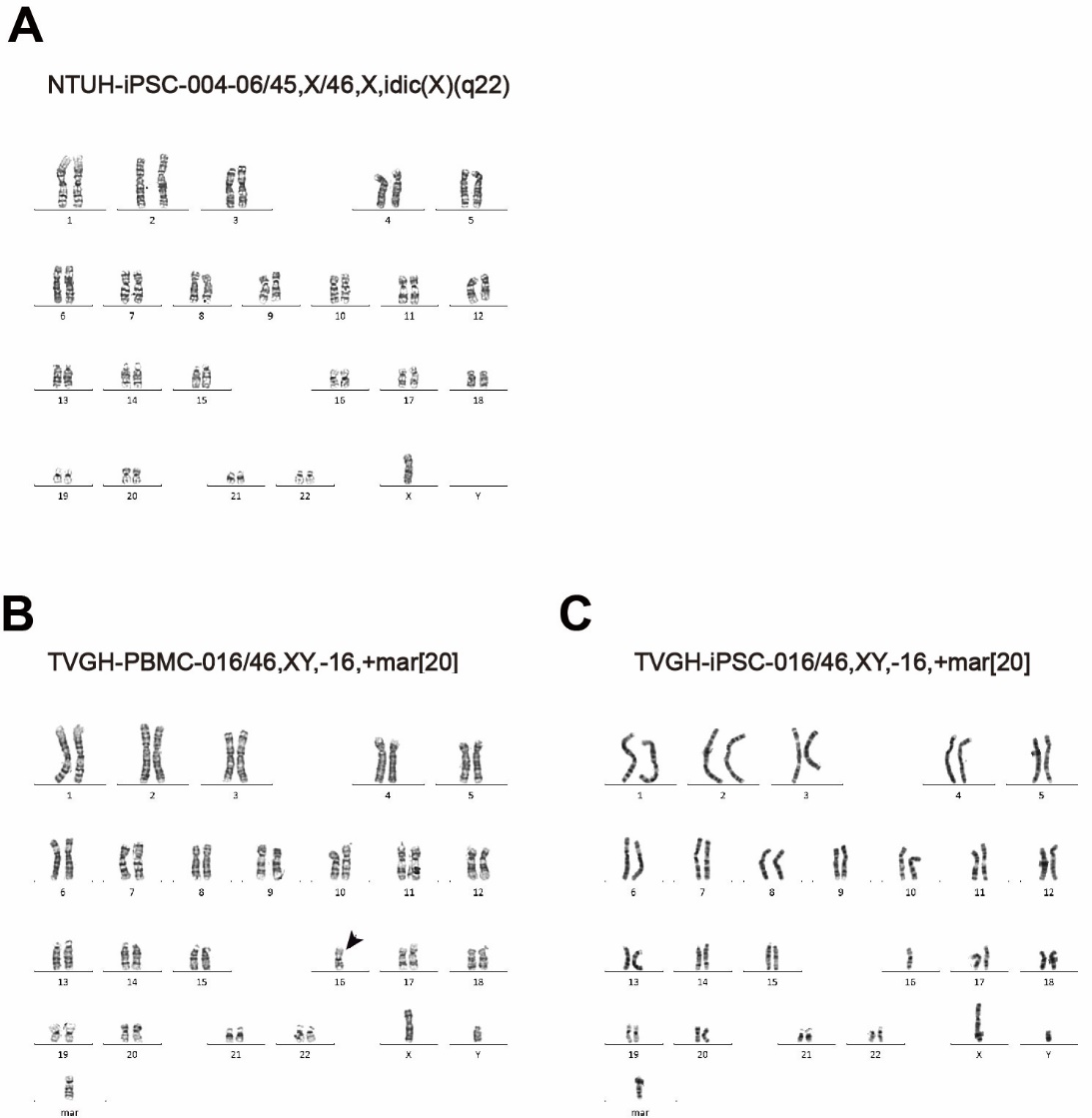


**Fig. S2 Karyotype analysis of the iPSC lines**. (A) Karyotype of premature ovarian failure iPSC, NTUH-iPSC-004-06 (Turner disease). (B) Karyotype of parental cells of TVGH-iPSC-016, arrow indicates rearranged chromosome 16. (C) Karyotype of TVGH-iPSC-016 (monogenic diabetes iPSC), arrow indicates missing chromosome 16.


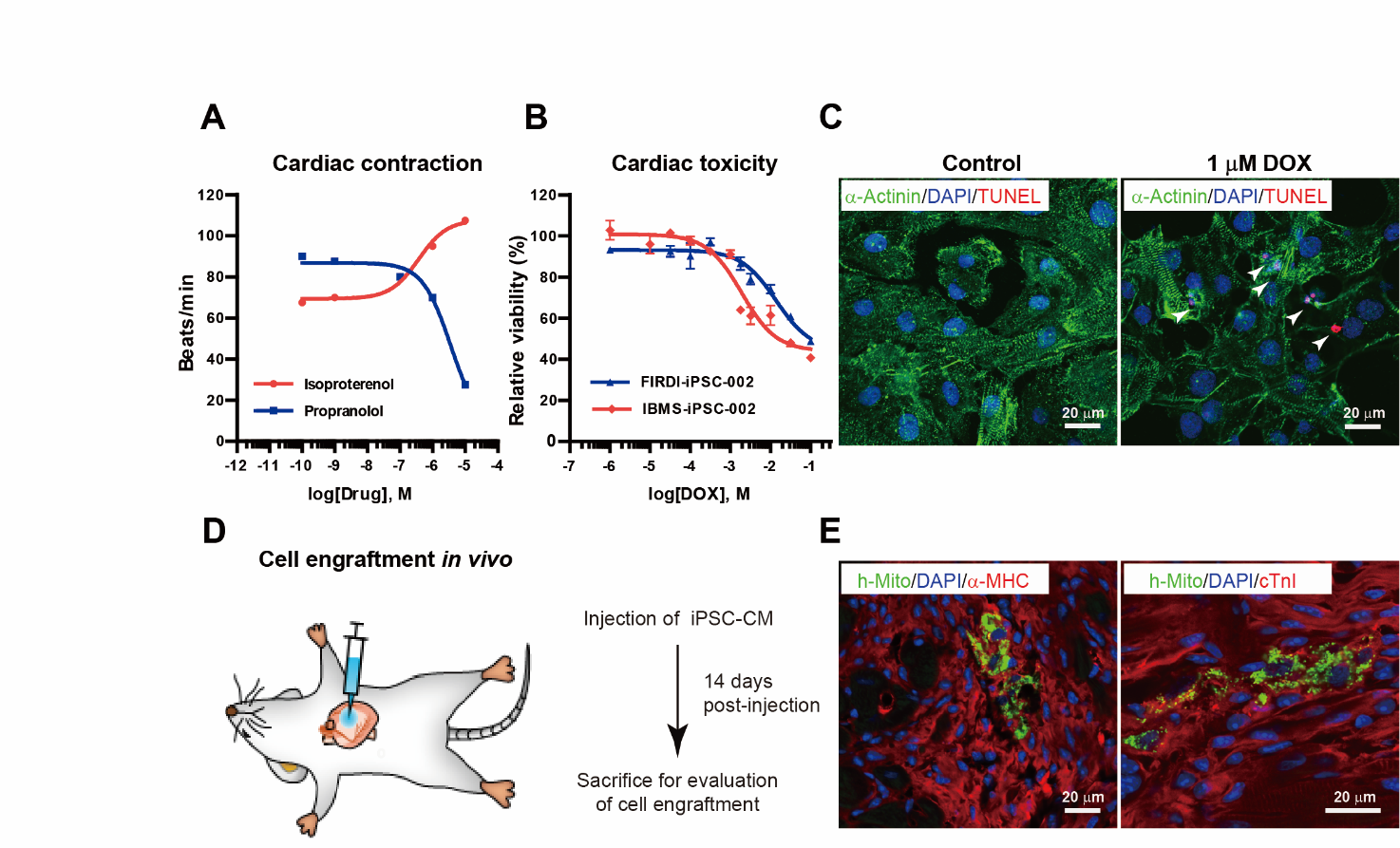


**Fig. S3 *In vitro* and *in vivo* functional evaluation of iPSC derived cardiomyocytes.** (A) Beating frequency (beats/min) of iPSC-derived cardiomyocytes under isoproterenol or propranolol treatment. (B) The dose-response relationship for doxorubicin (DOX) treatment of iPSC derived cardiomyocytes, as evaluated by TetraZ cell counting assay. (C) TUNEL assay showing cell death 24 hours after doxorubicin treatment. (D) Workflow of iPSC-CM engraftment in the mouse heart. (E) Immunostaining of the mouse myocardium engrafted with derived from human iPSC, showing engrafted of human iPSC-CMs. h-Mito: anti-human mitochondria antibody. Data are represented as mean ± SEM.

**Table S1 A List of Available Normal and Disease iPSCs in the Taiwan Disease iPSC Service Consortium Cell Bank**

**Table S2 The number of SNV, DEL, INS and MNV of iPSC-specific qualified variants**

**among iPSCs identified by GATK HaplotypeCaller**

**Table S3 List of iPSC-specific CNV loci**

**Table S4 Summary of iPSC-specific CNV loci among various iPSC lines**

**Table S5 List of genes at the "polymorphic" CNV regions strongly associated with the reprogramming process**

**Table S6 Primer list for RT-PCR**
